# What Are Good Situations for Running? A Machine Learning Study Using Mobile and Geographical Data

**DOI:** 10.3389/fpubh.2020.536370

**Published:** 2021-01-11

**Authors:** Shihan Wang, Simon Scheider, Karlijn Sporrel, Marije Deutekom, Joris Timmer, Ben Kröse

**Affiliations:** ^1^Informatics Institute, University of Amsterdam, Amsterdam, Netherlands; ^2^Information and Computing Sciences, Utrecht University, Utrecht, Netherlands; ^3^Human Geography and Spatial Planning, Utrecht University, Utrecht, Netherlands; ^4^Faculty of Health, Sports and Welfare, Inholland University of Applied Sciences, Amsterdam, Netherlands; ^5^Digital Life, Amsterdam University of Applied Sciences, Amsterdam, Netherlands

**Keywords:** mobile data mining, physical activity, running, machine learning, big data, environmental situations

## Abstract

Running is a popular form of physical activity. Personal, social, and environmental determinants influence the engagement of the individual. To get insight in the relation between running behavior and external situations for different types of users, we carried out an extensive data mining study on large-scale datasets. We combined 4 years of historical running data (collected by a mobile exercise application from over 10K participants) with weather, topographical and demographical datasets. We introduce weighted frequent item mining for the analysis of the data. In this way, we capture temporal and environmental situations that frequently associate with different running performances. The results show that specific temporal and environmental situations (hour in a day, day in a week, temperature, distance to residential areas, and population density) influence the running performance of users more than other situational features. Hierarchical agglomerative clustering on the running data is used to split runners in two clusters (with sustained and less sustained running behavior). We compared the two groups of runners and found that runners with less sustained behavior are more sensitive to the environmental situations (especially several weather and location related features, such as temperature, weather type, distance to the nearest park) than regular runners. Further analysis focused on the situational features for the less sustained runners. Results show that specific feature values correspond to a better or worse running distance. Not only the influence of individual features was examined but also the interplay between features. Our findings provide important empirical evidence that the role of external situations in the running behavior of individuals can be derived from analysis of the combined historical datasets. This opens up a large potential to take those situations specifically into consideration when supporting individuals which show less sustained behavior.

## 1. Introduction

Physical inactivity has been identified as a leading risk factor for poor health in modern society, as it can lead to serious physical and mental health problems (1, 2). In order to maintain a healthy lifestyle, people are advised to engage in a sufficient amount of physical activity on a regular basis [i.e., at least 150 min moderate-intensity activity every week for adults (3)]. However, a large group of individuals struggle with sustaining this healthy activity level. To illustrate, more than half of all Dutch residents in the Netherlands did not meet these guidelines in 2017 (4). Thus, searching for ways to promote sustained physical activity for less active individuals is a challenge (5, 6). Intelligent mobile systems can automatically and accurately track people's behavior and, based on this tracking, continuously intervene with a user to promote physical activity (7–10).

For this purpose, it is crucial to have insight in the context or situation of the user (11, 12). A situation can be defined as a snapshot of internal (e.g., fitness level, mood, etc.) and external variables (e.g., time, location, weather, etc.) of a person in a physical or conceptual environment (13). In daily life, situations can play an important role in determining the effectiveness of an intervention (14, 15). For instance, a reminder about physical activity sent during a meeting will likely be ineffective, no matter how persuasive its content might be. Also, environmental characteristics (situations) can play a role in influencing physical activity behavior (16). Both socioecological models (17) and cognitive models (18) explain how the physical environment can facilitate or discourage certain behaviors. Sufficient sport facilities, such as urban parks, might facilitate exercise whereas lack of facilities likely discourage physical activity (19).

Remarkable is that these situational barriers seem to have less impact on the individual when the activity habit is more established (20, 21). Hence, it is likely that the role of the physical environment on running behaviors differs between individuals in different stages of behavior change. Several studies demonstrated that the situational barriers of physical activity changes during the process of behavior change (20, 22, 23). Notably, all these studies used questionnaires to determine the relationship between situational barriers and human behavior. To examine whether this pattern also applies to not self-reported empirical data, we conduct a data-driven study with a combination of different datasets. We first set out to cluster individuals based on their activity levels. Then, the characteristics of situations suitable for running are examined for each cluster in more detail. Based on the findings of this study, tailored advise for running can be provided to mobile fitness application users to increase the persuasiveness of the application.

### 1.1. Previous Data Studies

A substantial body of literature addresses the situation of various physical activities by measuring the relationship between physical activity and contextual features (24), mainly including temporal, weather-based, topographical ones (25) and sometimes also including demographic ones (26). For instance, access to natural facilities and good road networks likely support physical activities (27, 28). Also, several studies found seasonal effects on the amount of physical activity, such as the association between colder and wetter seasons in Scotland with lower levels of leisure time physical activity (29).

However, most of those findings are based on research methods, such as interviews, questionnaires, and *in-situ* observations (30). As a consequence, studies have limited sample sizes, take into account only a limited amount of contextual variables, and often depend on the recall abilities of participants (31). Contrary, we now have the opportunity to collect large amounts of data about running behavior that was directly measured, due to the technological advances and the ubiquitous spreading of wearable devices and mobile phones (32, 33). This data was successfully used to examine the associations between physical activity behaviors in previous research. For instance, Jansen et al. (34) used a combination of a GPS sensor and accelerometer to determine the influence of the geographical environment on physical activity for different intensity levels, and Kim et al. (35) applied wearable devices to collect crowdsourced physiological data from pedestrians and analyze the features of walkable environment.

Furthermore, while smartphones deliver new opportunities to collect physical activity related data (36), several studies worked with mobile fitness application data to address the popularity of a physical activity in spatial and temporal contexts. Hirsch et al. (37) used data tracked by MapMyFitness to examine the patterns of different activities across geographic and temporal scales. In another study, Oksanen et al. (38) concentrated on geography-based heatmaps to understand popularity of locations for cycling, taking the diversity of cyclists into consideration. Focused on running activity, He et al. (39) used the Twitter data to understand preferred running times of Nike+ users, and clustered the users based on their different preferences. However, this study concentrated on temporal situations only. Later, Balaban and Tunçer (40) combined mobile application data from several resources to identify temporal and geographical situations for running and walking in Singapore, without taking the diverse preferences of individuals into consideration. In contrast to previous data studies in running activity, we considered a variety of features (covering weather-based, temporal, topographical and demographic situations) and investigated their association with running performance with respect to different types of users. As far as we can see, this is the first study done based on such substantially large datasets.

### 1.2. Our Research Objectives

In this article, we concentrate on modeling and investigating the relation between contextual situations and running behavior for different types of users. More precisely, we analyzed the correlation between situations when people start a run (X) and their performance in that run (Y), with respect to target users having different running behavior (Z). Details of X, Y, Z are indicated as follows:

- X: Situations: temporal, weather-related, topographical and demographical situations at the start point of a running activity;
- Y: Performance in one running activity: defined as the normalized distance of a run, with respect to an individual's ability;
- Z: Target users: individuals with different annual running patterns, including ones with less sustained behavior and ones with a sustained and regular behavior (denoted by *less active and active runners*).

In order to assess the three aspects in an integrated manner, we propose to use machine learning approaches. We first used a hierarchical agglomerative clustering algorithm to distinguish individuals with different annual running patterns. Then, we introduced weighted frequent item mining to extract the situations that are frequently associated with exceptional running distances (longer or shorter than an individual's average running distance). A mobile running dataset and two geographical datasets were used in this study, which contain 4 years of running history of over 10K users in the Netherlands. By combining and analyzing those datasets, we addressed and examined the following three research questions:

1. Which situational features are correlated with the running distance?
2. Do these features differ for people with different annual running patterns?
3. Under which situations do less active runners run longer or shorter than their average?

## 2. Methods

### 2.1. Data

In order to assess behavior of different types of runners in different situations, we need to categorize runners and measure the spatio-temporal context of a run. For this purpose, we introduce three datasets in this section. One large-scale dataset includes information of all participants' runs and was collected by a mobile exercise app. The two others datasets contain geographical data for enriching tracks with the spatial context of a situation. We present how these datasets were combined and processed to derive variables which describe the situation as well as the individual running behavior.

#### 2.1.1. Data Acquisition and Description

***Mobile fitness application data***.

This dataset was collected and provided by our cooperator MYLAPS^1^ using a mobile fitness application. This fitness app is launched in smart phones with either Android or IOS systems. All users agreed that their data could be used for scientific purposes. The data collection starts when the user clicks the “start exercise” button of the app and then continuously tracks all the data involved in a run in the background, until it gets terminated by the user in a comparable manner. In this way, we tracked historical running data of Dutch participants, mainly aged between 18 and 65, while using the app for physical exercises in their leisure time from 2013-03-23 to 2017-03-15.

In total, our dataset contains around 440K runs performed by over 10K users. Each run is identified by a unique running ID and grouped by a unique user ID (i.e., an anonymous code). For each run, a set of data records is collected summarizing total distance and runtime, as well as marking the time-stamp and weather information at the start point for the run. Moreover, a GPS tracker embedded in the mobile device provides GPS locations, which can be used to extract various geographical context features that might influence the activity. An example of the running data record used in this paper is given in Table 1. To protect the privacy of our participants, a data usage agreement was signed between researchers and the data provider. According to the agreement, no personal identification data was ever presented in our study, and all data processing and analysis were conducted following data privacy guidelines.

**Table 1:** An example of a data record in the mobile application dataset (due to data privacy guidelines, feature variables were randomly selected from different data records).

Activity ID	234014
User ID	11256
Total distance	9,870 m
Timestamp at start-point	2016-05-05 10:31:18.0000000
GPS at start-point	(53.20317, 5.82213)
Weather type at start-point	cloudy
Temperature at start-point	11°C
Wind type at start-point	light wind
Humidity type at start-point	middle humidity

***Geographical data***.

In order to model the environmental context of a runner's start location, we made use of two geographical datasets covering the *topography* and the *demographics* of the neighborhood. We chose this because prior studies used topographical properties (27) (such as the surrounding landuse mix) as well as socio-demographics (26) (such as population density) as descriptors of context.

**Figure 1:**
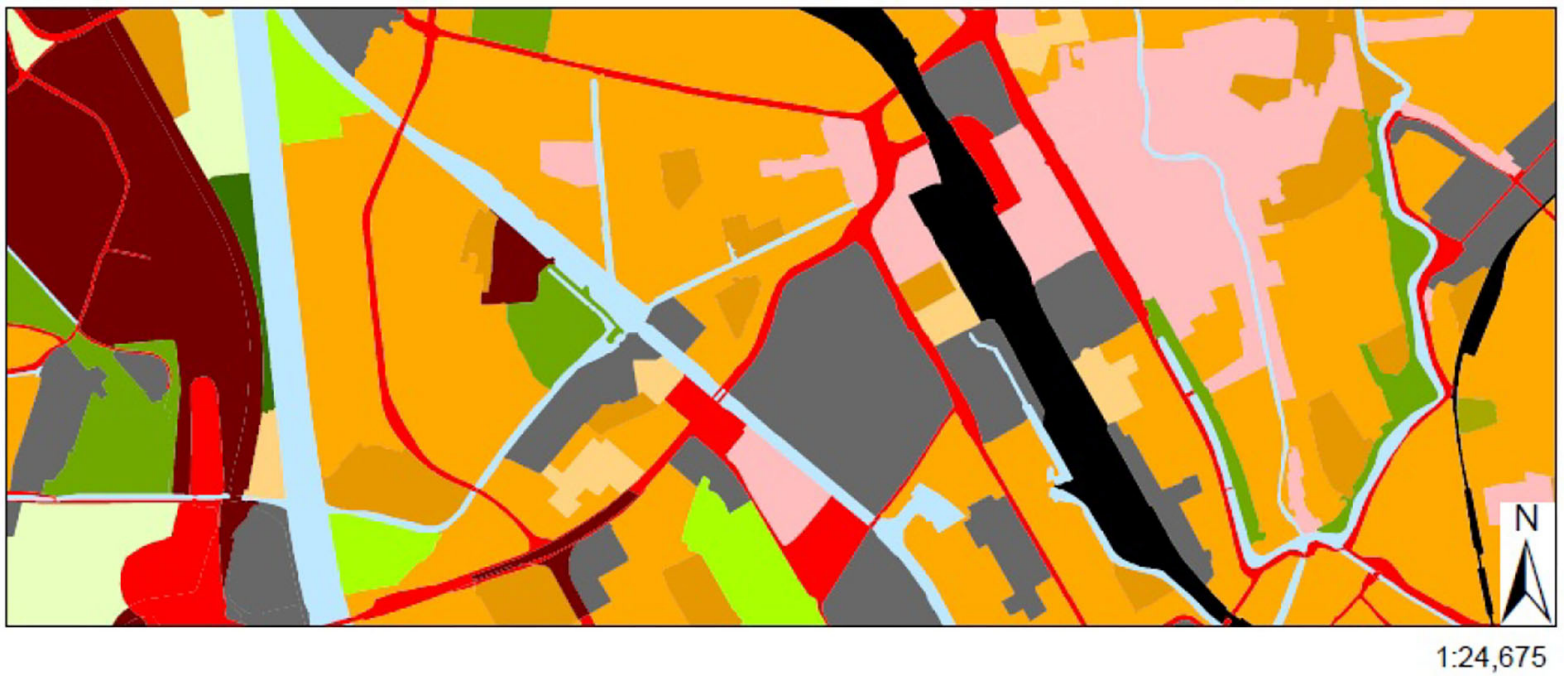
The landuse data is used for assessing the spatial running context. Here a map for the city of Utrecht is shown as an example (Source: CBS 2012), where areas in different colors represent different kinds of landuse classes.

For topographic information we used *landuse* data from the Dutch Centraal Bureau voor de Statistiek (CBS), namely *Bestand Bodemgebruik* (BBG 2012)^2^. The data contains a collection of spatial regions labeled with various landuse classes which are assumed to apply homogeneously across these regions (an example of this map for Utrecht is shown in Figure 1). We reclassified the given CBS classes, then identified nine categories that are deemed relevant for the landscape context of physical activity (27, 28, 41), including parks, sports areas, recreations areas (like camping, animal/theme park, and playground), forests, water areas, agriculture areas, traffic areas, residential areas, and central business district. For instance, in Figure 1, locations in blue correspond to water, while red ones are traffic related areas.

For the demographical data we used the CBS statistics (Kerncijfers) data source for statistical neighborhoods (Wijk- en Buurtkaart 2015)^3^ to capture the demographic environment of a neighborhood for running. Neighborhoods are statistical areas of highest resolution in the Netherlands, and Kerncijfers include statistics about their inhabitants and households. Thus, the demographic features include variables that describe why a neighborhood might be perceived as an attractive base for a certain activity (like running). In summary, we used the following 4 attributes: density of people (per km^2^), percentage of one person households (per km^2^), percentage of households without children (per km^2^) and percentage of inhabitants over 65 years (per km^2^).

#### 2.1.2. Data Cleaning and Processing

**Figure 2:**
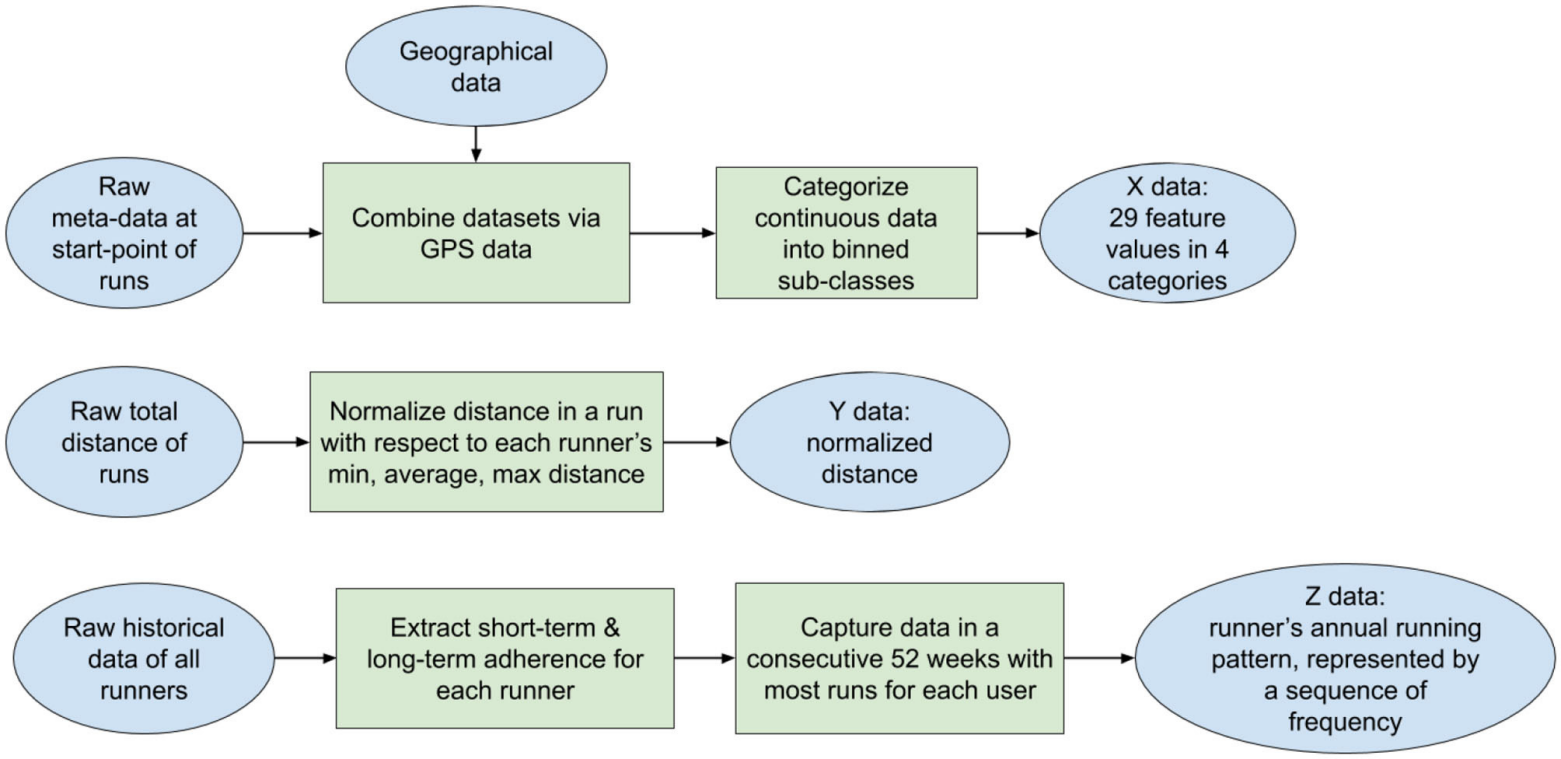
The workflow of data processing. The raw datasets are transformed into three elements X, Y, Z for data mining.

We firstly filtered out raw running samples with missing or erroneous values (for instance, runs with a total distance <100 m were removed), which accounts for <5% of total runs. Then, following the workflow in Figure 2, we transformed the raw running data into three elements involved in the data analyses (X, Y, Z defined in section 1).

***Feature extraction and binning***.

In this study, 29 features that may correlate with the running activity (30) were extracted from the combined data. A list of extracted features and their corresponding type is presented in Table 2. In summary, four categories of features are considered: temporal ones that define the calendar time, weather-related ones that define weather conditions, topographical ones that define the physical properties of a location, and demographical ones that describe the inhabitants surrounding a location.

**Table 2:** A list of extracted features with their situation type.

**Category and description of feature**	**Category and description of feature**
Temporal	8. Distance to nearest residential (rsda)
1. Time in a day (hour)	9. Distance to nearest business area (cbd)
2. Weekday in a week (weekday)	10. Percentage (%) of traffic in 1*km*^2^ (trfc-cov)
3. Month in a year (month)	11. % of water in 1*km*^2^ (wtr-cov)
Weather	12. % of recreation in 1*km*^2^ (recr-cov)
1. Temperature	13. % of forest in 1*km*^2^ (frst-cov)
2. Weather type (weather)	14. % of sports in 1*km*^2^ (sports-cov)
3. Wind type (wind)	15. % of parks in 1*km*^2^ (park-cov)
4. Humidity type (humidity)	16. % of agriculture in 1*km*^2^ (agri-cov)
Topographical	17. % of residential in 1*km*^2^ (rsda-cov)
1. Distance to nearest traffic (trfc)	18. % of business areas in 1*km*^2^ (cbd-cov)
2. Distance to nearest water (wtr)	Demographical
3. Distance to nearest recreation (recr)	1. Population density in *km*^2^ (population density)
4. Distance to nearest forest (frst)	2. % of inhabitants over 65 in *km*^2^ (over 65)
5. Distance to nearest sports (sports)	3. % of one-person households in *km*^2^ (one person)
6. Distance to nearest park (parks)	4. % of households without children in *km*^2^ (no child)
7. Distance to nearest agriculture (agric)	

For our temporal features, calendar time was derived from the “timestamp at start-point,” while weather-based features were given directly in the collected mobile application data. With the GPS point of the start location of the running activity, we could infer its topographical and demographical properties by incorporating the data resources described in section 2.1.1. First of all, the *topographical context* of a start location was captured based on two different spatial properties:

1. *Euclidean distances* from the start location *to the nearest region* of a given landuse class, for each of the 9 classes. This captures the probability of a runner to interact with or *access* this landscape type, either in terms of visibility or in terms of using it as a support surface for running (distance = 0). This influences whether a certain landuse type is accessible or not.
2. *Percentage of spatial coverage* of a given landuse class within a 1 km^2^ rectangle around each start location, for each of the nine classes. This captures the *spatial density* and dominance of a landuse class in the running environment, which influences the perceived layout of the location.

**Figure 3:**
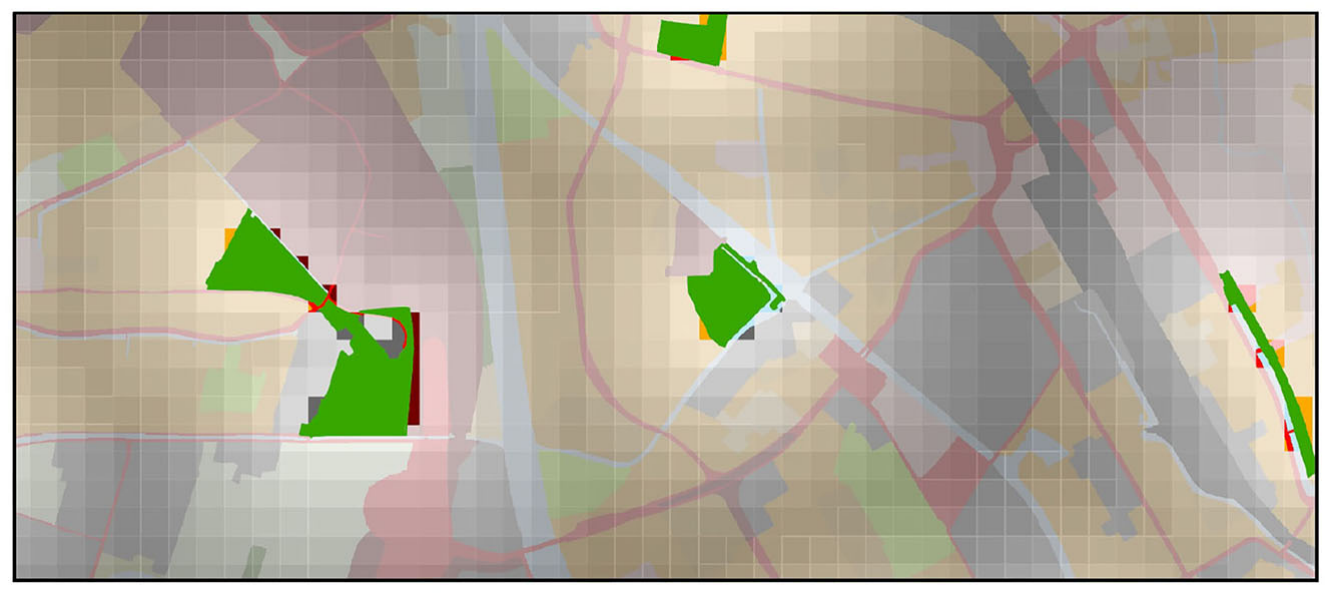
An example of some park areas in Utrecht with computed distance rasters.

For both properties, we first generated a regular grid of 100*100 m cells over the entire Netherlands that was used to enrich a given location in the runner data^4^. To capture the distance context we computed the linear spatial distance (in meter) from each grid cell to the nearest region for each of the selected landuse categories (see the example for “parks” in Figure 3). To determine the coverage context, we computed the number of grid cells that are covered by a landuse area of the respective type in a 10*10 cell rectangle window around each location. Since the number of cells in this rectangle is 100, this value corresponds to the percentage of coverage. Similarly, for the *demographic context*, we spatially queried the demographics dataset about the statistics of the neighborhood in which the start of running is located (using a point-in-polygon query) and added the corresponding attributes to the runner data.

Lastly, in order to apply weighted frequent item mining (presented in section 2.2.2) to the features denoting continuous variables, we grouped their values into a series of intervals by *data binning*. In this study, the topographical and demographical bins were determined based on the prior distribution of the geography over the entire Netherlands. More precisely, we computed distances, coverages and demographic values for each raster cell in the Netherlands and then generated 10 quantiles [0%], [0, 10%], [10, 20%], … over the cell distribution to determine bin boundaries for the corresponding features. This helped us better interpret the values, telling us whether they occur seldomly or not. The value categories of all features used in our data analysis are listed as follows:

- time in a day: eight equal bins from hour 0:00 to hour 24:00.
- weekday in a week: seven categories from Monday to Sunday.
- month in a year: 12 categories from January to December.
- temperature (Celsius): five equal bins in [−10, 30] degree.
- weather type: nine categories, being sunny, half cloudy, cloudy, rainy, windy, thunderstorm, snow, hail, and mist.
- wind type: five categories, being windless, light wind, moderate wind, hard wind, and storm.
- humidity type: three categories, being low humidity, moderate humidity and high humidity.
- distance to the nearest nine categories of landuse: 0 m, (0–10% quantile] m, (10–20% quantile] m, …, (90–100% quantile] m.
- population density per km^2^: 0, [1, 13], [14, 25], [26, 55], [56, 152], [153, 2,132], [2,133, 7,623], [7,624, 13,114], [13,115, 18,605], [18,606, 28,370].
- percentage of nine categories of landuse coverage and 4 demographic densities: 0 percentage, (0–10% quantile] percentage, (10–20% quantile] percentage, …, (90–100% quantile] percentage.

So every run has a feature vector consisting of 29 categorical feature values. We call any combination of these feature values a *situation* (X) of the run.

***Running distance normalization***. In addition, we measured *running performance* (Y) based on the “total distance”^5^ in Table 1. To account for the differences in running capabilities between different users, the running distance was normalized with each user's personal statistics. To illustrate, imagine the following situation. Some user might run 5 km on average, whereas another runs on average 15 km. When both of these users run 10 km in one record, this performance is relatively good for the first user, but worse than average for the second one. The normalization of distance is given in Equation (1), resulting in a scale ranging [−1, 1]. By doing so, a distinction can be made between users' different behavior in each running activity.

(1) $$\begin{array}{l} y_{i}^{j^{\prime }}=\frac{y_{i}^{j}-average\left({\bar{y}}^{j}\right)}{max\left({\bar{y}}^{j}\right)-min\left({\bar{y}}^{j}\right)} \end{array}$$

where $y_{i}^{j}$ is an original value of distance in one run *i* of user *j* and $y_{i}^{j^{\prime }}$ is its normalized value, given *min*(ȳ^*j*^), *average*(ȳ^*j*^), and *max*(ȳ^*j*^) are the minimal, mean and maximal value of distances in all runs performed by user *j*. The normalized value now tells us whether a runner performed above or below his or her average running distance, as well as how close to his/her best and worst performances.

**Figure 4:**
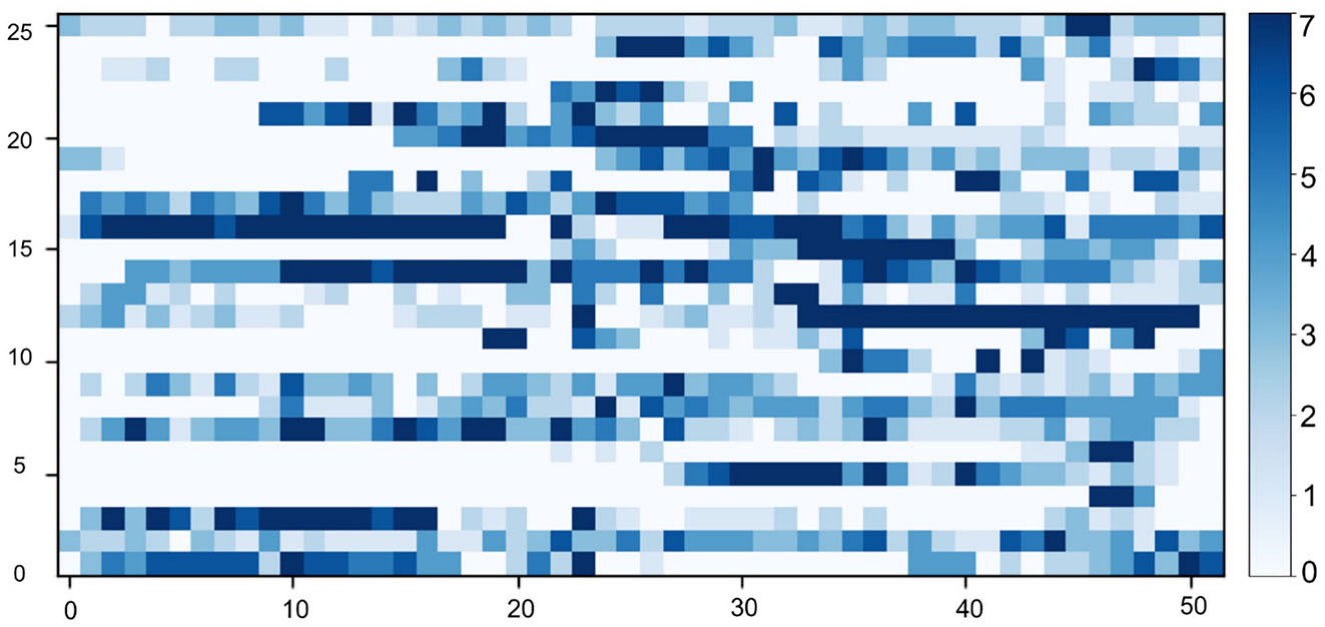
A visualization of the annual running pattern of 25 random users, where the y-axis represents the user and the x-axis represents 52 consecutive weeks of a year. The blue scale shows the running frequency of a user in a week.

***Annual running pattern extraction***. In our study, runners (Z) were characterized based on the temporal running pattern to distinguish their sustainability in running activity. This was defined based on both short-term running adherence (running frequency in a week) and long-term running adherence (sequence of running weeks during a year). We extracted the temporal running pattern of all users into a matrix of running frequency in the following manner. We started by filtering users with <10 runs in 4 years, since they contributed limited runs in our dataset and might bring potential data bias into our analysis (for instance these people might give up using the app after few runs, as most of them only have records within the first 2 weeks). We then processed the running frequency per week for each runner, followed by analyzing his/her long term adherence over 52 weeks (one year interval). For each runner, a sliding window mechanism was applied to extract consecutive sequences of 52 weeks and to select the most active one (i.e., the window with the most runs). To this end, we formatted the historical running data of each user as a sequence of weekly-based running frequency. In this way, we built a data matrix with 5,346 distinct users and their running activities in 52 weeks (i.e., *D* ∈ ℝ^5,346×52^), where users and weeks are represented in rows and columns, respectively. This matrix covers around 270K running activities. We plotted a matrix with random 25 users in Figure 4.

### 2.2. Data Analysis

**Figure 5:**
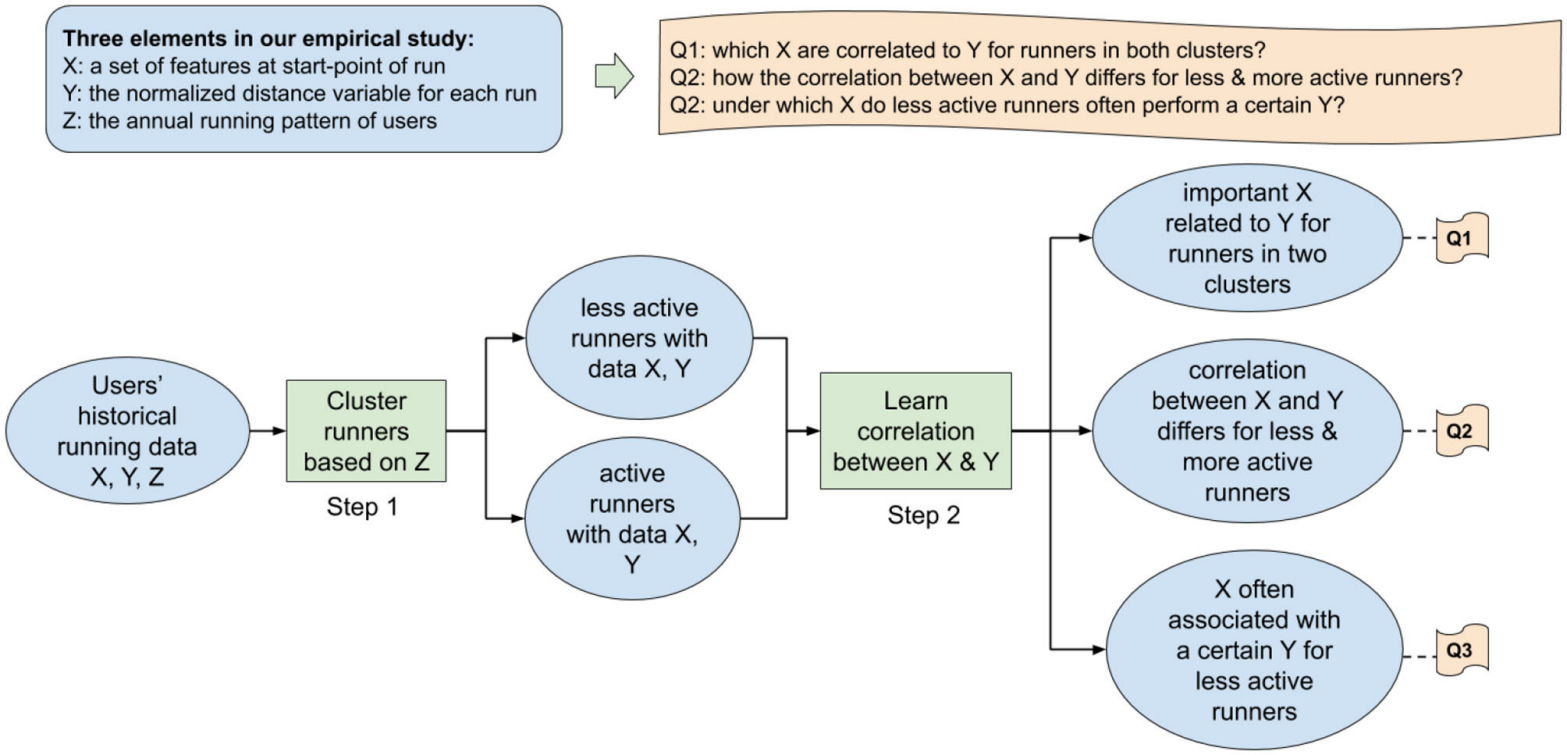
The workflow of our empirical data analysis.

In this section, we present how we applied machine learning to explore the proposed research questions. The workflow of our empirical data analysis is presented in Figure 5. We first clustered runners based on their annual activity patterns to distinguish between regularly active and less active runners (referring step 1 in Figure 5). Next, we investigated the correlation between situations and the normalized distance of runs using *weighted frequent item mining* (referring to step 2 in Figure 5). The methodology of clustering and weighted frequent item mining are described with more details in sections 2.2.1 and 2.2.2, respectively.

#### 2.2.1. User Clustering

In step 1 of Figure 5, we employed hierarchical agglomerative clustering to group users based on their annual running patterns extracted in section 2.1.2. This is a standard “bottom-up” method without prior setup for the number of clusters. The clustering algorithm starts with pairing users with similar annual running pattern, then gradually grouping them into bigger clusters. In this procedure, it reveals the overview of hierarchical structure for all users, which provides insight on the obtained clusters. Thus, our approach allows the adaptation of clustering threshold based on the domain knowledge. This is important in our case since a variety of similar annual running patterns could be captured in each user cluster. We developed our hierarchical clustering method as follows.

First of all, the similarity between each pair of data entities (i.e., the sequence of running frequency for each user) was measured using dynamic time warping (abbreviated as DTW). It identifies the “optimal warping path” between paired data. Instead of only comparing an individual value at a certain time index, DTW compares two paired data series by transforming their indices over the entire time period (42). This overcomes differences in timing. In our analysis, a python library^6^ was used to calculate the dynamic time warping distance.

Furthermore, we applied the Ward variance minimization algorithm to estimate the similarity of paired clusters and merged ones that are close by minimizing the variance within the newly formed cluster. More precisely, given two clusters *C*_*x*_ and *C*_*y*_ to be merged (where *C*_*x*_ is a new joint cluster with two sub-cluster *C*_*i*_ and *C*_*j*_, and *C*_*y*_ is an unused cluster), the Ward's distance between *C*_*x*_ and *C*_*y*_ is presented by the following *recursive* equation:

(2) $$\begin{array}{l} d\left(C_{x},C_{y}\right)=\sqrt{\frac{n\left(C_{i}\right)+n\left(C_{y}\right)}{n\left(C_{x}\right)+n\left(C_{y}\right)}d{\left(C_{i},C_{y}\right)}^{2}+\frac{n\left(C_{j}\right)+n\left(C_{y}\right)}{n\left(C_{x}\right)+n\left(C_{y}\right)}d{\left(C_{j},C_{y}\right)}^{2}+\frac{n\left(C_{y}\right)}{n\left(C_{x}\right)+n\left(C_{y}\right)}d{\left(C_{i},C_{j}\right)}^{2}} \end{array}$$

where *n*(*C*) is the number of elements in a cluster and *d*(*C*_*i*_, *C*_*j*_) is the Ward's distance of cluster *C*_*i*_ and *C*_*j*_. In this way, small clusters are growing into bigger ones and eventually form a dendrogram. This dendrogram indicates the hierarchical structure of all users, while user clusters can be obtained using an adjustable threshold measuring their diversity.

#### 2.2.2. Weighted Frequent Item Mining

Next, we introduce the weighted frequent item mining approach (step 2 of Figure 5). Using this approach, we aim not only to study the relation between extracted features and running distance, but also to explore the complex interplay of various features (by capturing the combinations of various feature values that frequently associated with either longer or shorter running distance). Frequent item mining (abbreviated as FIM) is an important subfield in data mining, which is commonly used to discover interesting patterns from data based on their frequency. Weighted frequent item mining extends the traditional FIM problem by considering a weight-based constraint. By giving each data entity a weight, weighted FIM captures the importance, interest or profit of an individual data sample toward some goal (43). In our case, this common goal is the normalized running distance.

Let *I* = {*i*_1_, *i*_2_..., *i*_*n*_} be a set of distinct items in a transaction dataset *TD* = {*T*_1_, *T*_2_, ..., *T*_*m*_}, where the transaction *T*_*m*_ is a subset of items in *I*. *T*_*m*_ has a unique identifier called *TID*_*m*_ and a weight *w*_*m*_ showing its importance in *TD*. In this analysis, while a transaction *T*_*m*_ is the feature vector of a running activity (including 29 feature values extracted in section 2.1.2), an item *i*_*n*_ represents a specific value (for instance *Sunday*) of an feature (for instance “weekday”). Moreover, the normalized distance variable of this run is used as weight *w*_*m*_, ranging over the set of real numbers from −1 to 1 (i.e., ℝ ∈ [−1, 1]). Thus, given a weighted transaction dataset (i.e., running data of users) and a minimum support threshold (notated as σ), we aim to find the complete set of frequent combinations of items (i.e., situations), which is defined as our *weighted frequent item mining problem*.

**Figure 6:**
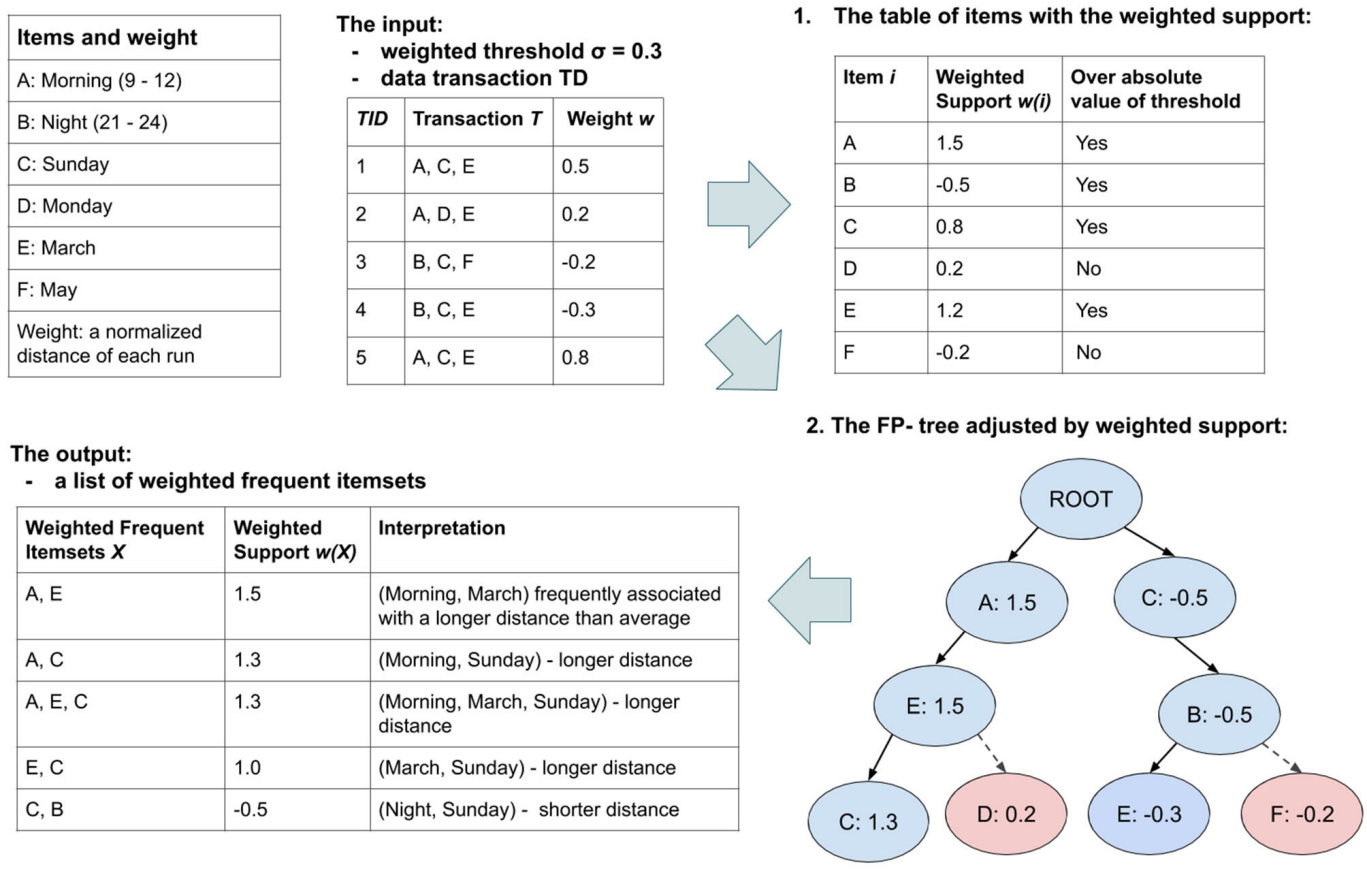
An illustration of the weighted FP-Growth algorithm pipelines, based on an example of our running activity data. Given weighted data *TD* and threshold σ, the weighted support of all items is firstly calculated and listed in a table. A prefix tree is then generated for efficiently acquiring frequent itemsets whose absolute value of support is above the threshold. More details about how to generate, prune and traverse the prefix tree can be found in Han et al. (44).

In our study, we solve this problem by extending a classical FIM algorithm, namely frequent pattern growth algorithm (44) (known as FP-Growth). The algorithm particularly performs efficiently on large-scale datasets like ours. Using our running activity data as an example, we illustrate the process of weighted FP-Growth algorithm in Figure 6. In such pipelines, we adjusted both the item head table and FP-tree by our defined weighted supports. The weighted support of an item *i*_*n*_ in TD equals the summarized weight of all transactions in TD containing this item, defined as *w*(*i*_*n*_) = ∑*w*_*i*_, for ∀*T*_*i*_ ∈ TD, where *i*_*n*_ ∈ *T*_*i*_. Similarly, the weighted support of itemset *X* equals the summarized weight of all transactions in TD containing itemset *X*, defined as *w*(*X*) = ∑*w*_*i*_, for ∀*T*_*i*_ ∈ *TD*, where *X* ⊆ *T*_*i*_. For either item *i*_*n*_ or itemset *X*, if the absolute value of its weighted support is lower than the threshold, it is pruned from the frequent itemsets (like red node D,F in the FP-tree of Figure 6).

In this manner, we addressed the relations between situations and running performance using the weighted support of frequent itemsets (i.e., situations). Thus, the *weighted support* of a situation is a summary of the running distance of all runs performed under this situation. Moreover, while only itemsets whose absolute value of weighted support is above the given threshold are selected, it helps us to capture the most frequent and interesting situations. For instance, in the example of Figure 6, the frequent itemset *(Morning, March, Sunday)* has a relatively high weighted support, indicating this situation often happened at the start-point of better performed runs. On the opposite, *(Night, Sunday)* is more frequently associated with relatively shorter running distance.

## 3. Results

In this section, the results of data analysis are presented following the workflow shown in Figure 5. We start with the clusters of runners based on their different annual activity patterns. Then we discuss the relationship between different features and the running distance for less active runners and active runners. Finally we concentrate on the less active runners and present their different behaviors under various situations.

### 3.1. Clustering Runners by Annual Running Pattern

**Figure 7:**
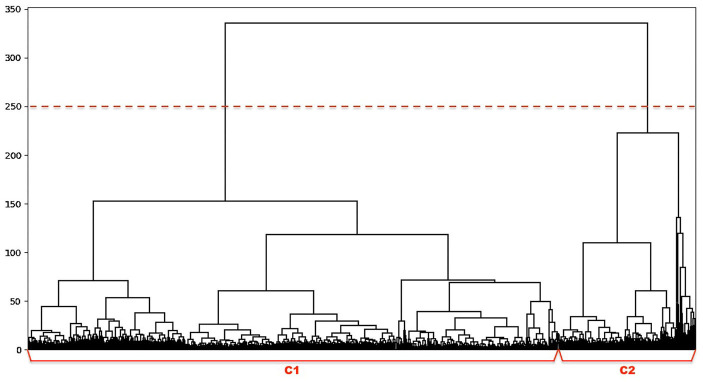
The dendrogram of hierarchical clustering of runners, where each unit on the x-axis represents one user and their diversity in activity is measured by the y-axis.

Firstly, groups of runners were captured by the hierarchical clustering algorithm in step 1 of Figure 5. The overall structure of 5,346 users was derived as the dendrogram in Figure 7. According to the figure, two runner clusters C1 and C2 can be determined based on the diversity of users' annual running patterns (using a threshold at 250).

**Figure 8:**
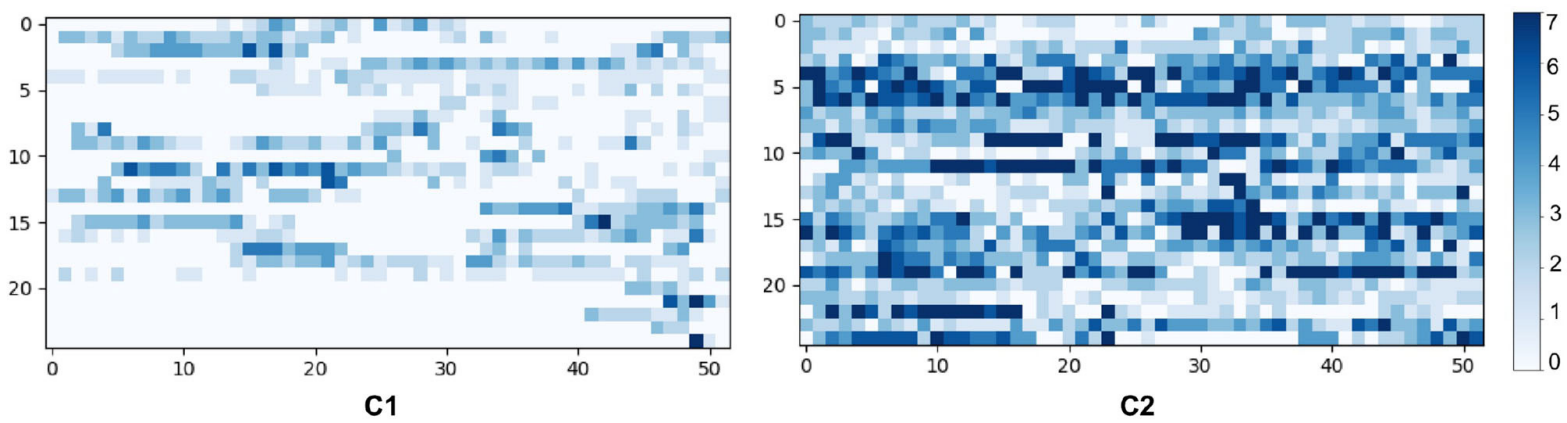
Temporal patterns of 25 random users in each cluster, where the y-axis represents the user and the x-axis represents the week of a year. The blue scale implies the running frequency of a user in a week.

To evaluate our findings, we visualized the characteristics of the clusters by randomly selecting 25 users from each cluster in Figure 8. According to the visual analysis, we can clearly discriminate between runners based on their running adherence in both short-term (weekly) and long-term (yearly). These patterns can be summarized as follows:

- **C1**: 4,257 users on average perform about 34 runs annually, either loosely throughout a year or spreading in a partial period of the year.
- **C2**: 1,087 users on average performing about 94 runs consistently over a year.

In the following sections we make use of these two clusters to answer our three research questions.

### 3.2. Relating Features to Running Distance

Applying the weighted frequent item mining in the running data of C1 and C2 user group, respectively, we investigated the contribution of the various features to the running distance (referring to the results acquired by step 2 in Figure 5). We considered all 29 features presented in Table 2 and examined their individual associations with the normalized running distance. The strength of such relation is measured by the variability of weighted support over all possible values that occur in a feature. For instance, while *Sunday* is a specific value of the feature “weekday,” the weighted support of *Sunday* is a sum of normalized distances from all runs performed on Sundays. Thus, the relation between feature “weekday” and running performance is captured by the variance of weighted support over different weekdays. A larger variance indicates that the different values of this feature (in this case weekdays) tend to cause more differences in terms of running distance.

**Figure 9:**
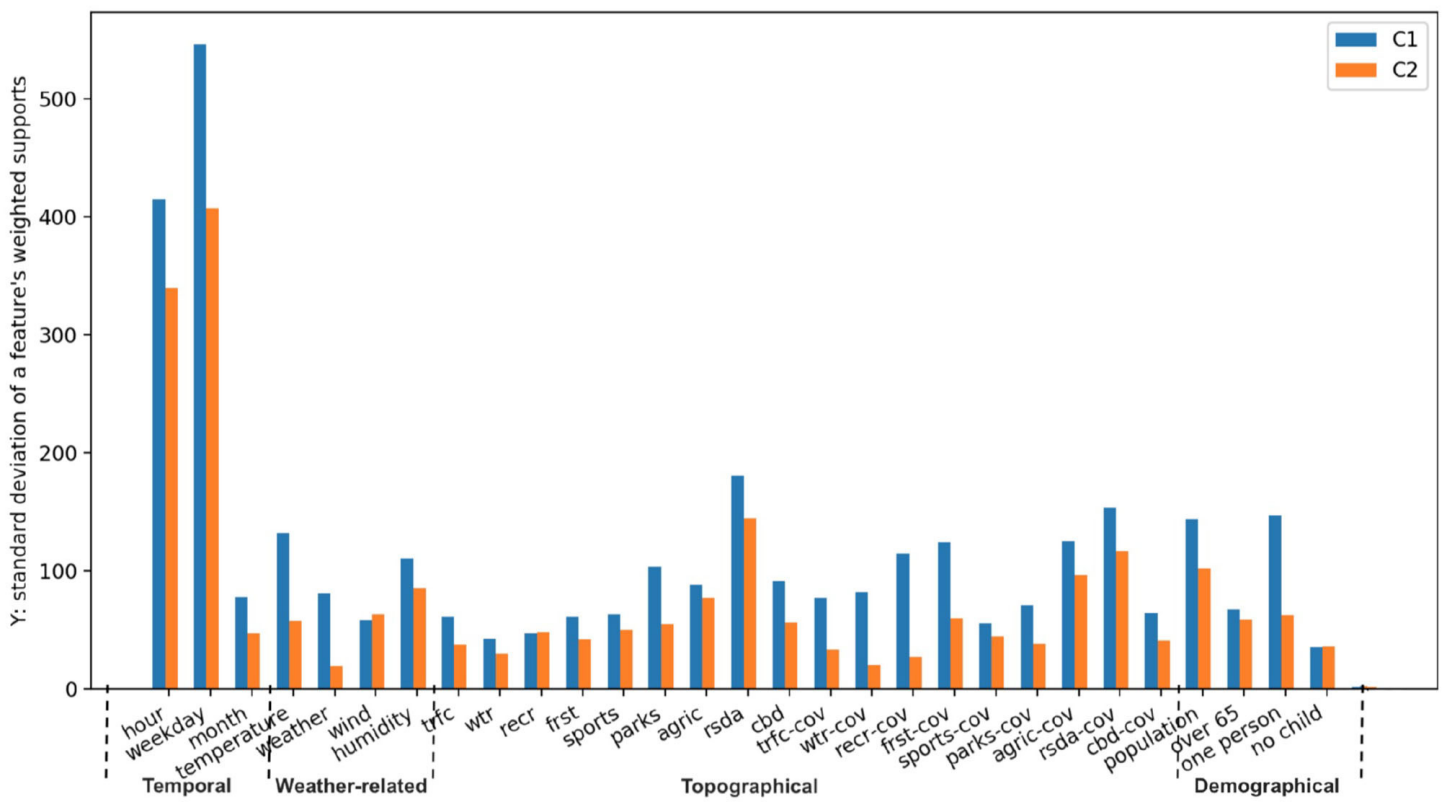
The comparison between C1 and C2 user group for all 29 features. The abbreviated names of features at x-axis are explained in Table 3, where y-axis represents the standard deviation computed from a list of weighted supports corresponding to all values of each feature.

In Figure 9, we present the standard deviation computed from the list of weighted support over all values for each feature. Considering both groups of runners, some features have a larger standard deviation than the others, such as “hour in a day,” “day in a week,” “temperature,” “distance to residential areas,” and “population density.” Hence, it suggests that these temporal and environmental variables influence the running distance of people to a larger extent.

### 3.3. Differences Between the Two Clusters of Runners

In Figure 9, we also show how the relation between chosen features and defined running distance differs for different runners. We noticed the standard deviations of almost all features in C1 are larger than those in C2 (except for “wind type”). This observation indicates that our chosen features generally have a greater influence on runners in C1 than the ones in C2. In other words, whereas the performance of less active runners appears to be more sensitive to these features, regularly active runners seem to ignore many of them. This is especially true for certain features related to weather and location, including “temperature,” “weather,” “distance to the nearest park,” “coverage of recreation/water,” and “percentage of residents living alone.”

### 3.4. Relating Situations to Less Active Runners' Running Distance

To get more insight into the variances of different features, we analyzed the *distribution* of relative running distance over different feature values (i.e., situations). In this section, we focus on the less active runners because their variance is larger and analysis results can potentially be used to obtain a more active running behavior for such runners. Using the results from the weighted frequent item mining approach, we first present the distributions for single features, then illustrate combinations of values from multiple features. In this way, we answered the research question: under which situations do less active runners run longer or shorter than their average.

#### 3.4.1. Situations From Single Features

**Figure 10:**
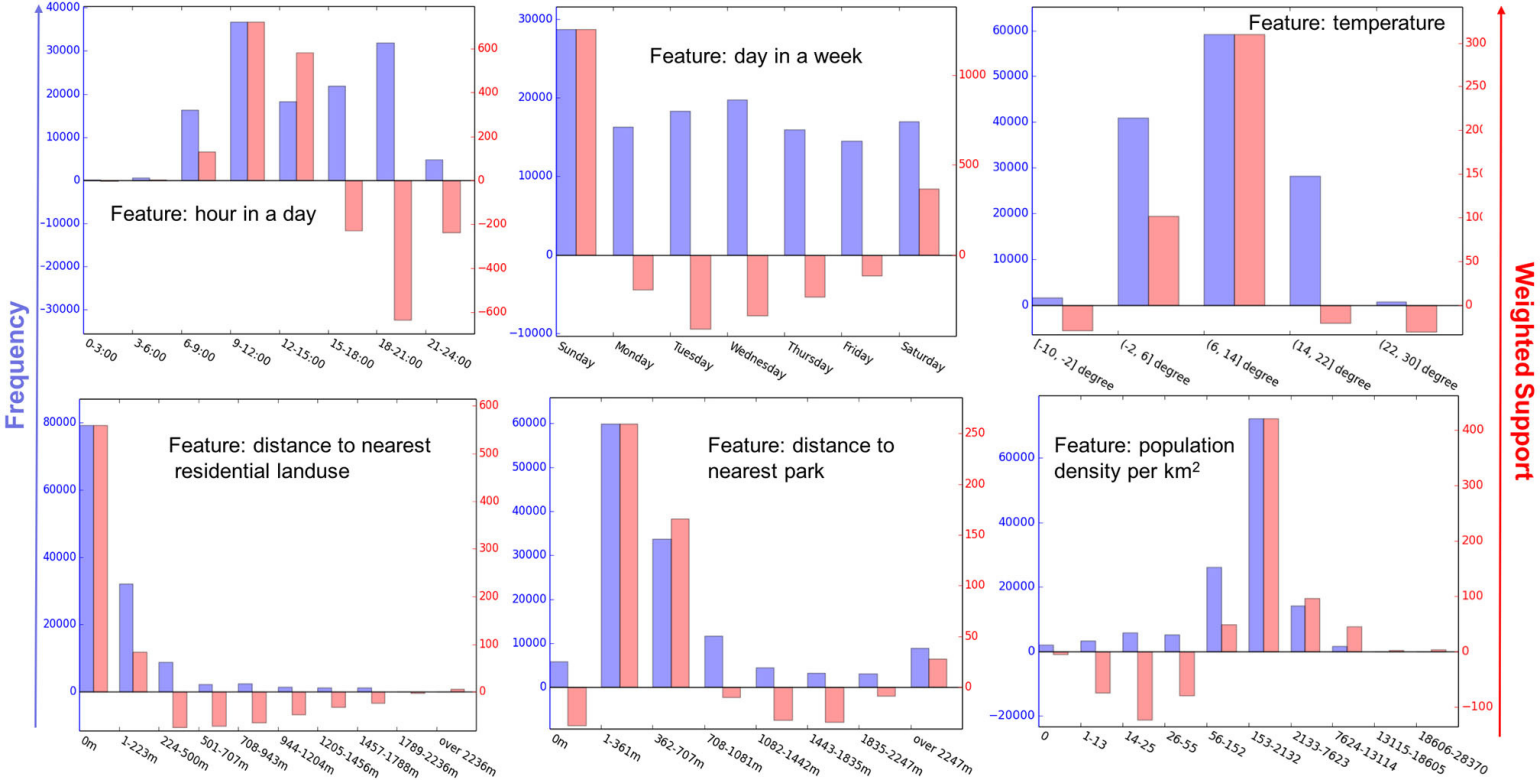
The histogram of weighted support (in red) and frequency (in blue) of all values for six features. Here, the frequency value indicates how often runs are performed under a certain situation (i.e., a feature value), while the weighted support value implies how often the situation can be associated with either a longer or shorter running distance than average (correspond to a positive and negative value, respectively).

We first studied the distribution of normalized running distance over all possible values of the features in Figure 9. The distributions of six representative features are plotted in Figure 10, including “hour in a day,” “day in a week,” “temperature,” “distance to nearest residential landuse,” “distance to nearest park,” and “population density.” These features were selected because they have the highest variances in each feature category (referring to Figure 9). To get more insights of the feature values, we also computed how many runs are performed under each feature value in the analysis (known as running frequency and shown in color blue in Figure 10).

In Figure 10, we can see that certain situations show significant associations with running frequency and running distance of less active runners. For instance, mornings (9:00–12:00), Sundays and moderate temperatures (between 6 and 14°C) attract less active people to run often and far. Considering the physical environment, less active runners often start nearby parks (about 350 m), and they tend to perform above average when they do so. Another very obvious pattern is that those people most frequently start their run and perform above average within or next to residential areas (see Figure 10). The population density plays an important role in demographical features. People generally perform best in neighborhoods with moderate population density (around 150 people per km^2^), and tend to run less far if the neighborhood gets less or more densely populated.

Furthermore, we observed an inconsistency between frequency and performance of less active runners. The popular situations with a high frequency of running are not always associated with better performance (a longer running distance than individual's average). For instance, for the feature “hour in a day,” runners show the best performance around 9:00–12:00, and the worst one around 18:00–21:00, although people do run frequently in both time periods. We also found that runners appear to run frequently and rather far on the weekend days, in particular Sundays. In contrast, people tend to under-perform on weekdays, even on Wednesdays, which is the second frequently chosen weekday.

#### 3.4.2. Situations From Multiple Features

Next, we analyzed the distribution of normalized running distance for situations with a combination of values from multiple features. To avoid the large amount of possible combinations among feature values, we separately looked at situations in four categories (i.e., combining values of features in each kind of category only). We then picked and presented several situations in each category in Table 3, which have the highest or lowest weighted support and cover a large number of features. In a nutshell, under situations on the left column of Table 3 (e.g., *9:00–12:00 on Sunday of February*), less active runners tend to run a longer distance than their average. On the contrary, situations on the right column are often associated with running distance below the average.

**Table 3:** Situations in four categories with a large absolute value of the weighted support.

**Category**	**Often with distance above average**	**Often with distance below average**
Temporal	*(Hour 9–12:00, Sunday, Feb./Sep./Nov.)*	*(hour 18–21:00, Tuesday/Wednesday)*
	*(Hour 12–15:00, Sunday, Sep./Nov.)*	
Weather-related	*([6,14] degree, half cloudy, light wind, middle humidity)*	*(Rainy, middle wind, high humidity)*
Topographical	*[0 m from residential area, 0–361 m (0–10% Q) from parks]*	*(0 m from agriculture, 0% coverage of recreation, 0% cov. of residential)*
	*[0% cov. of forest, 510–728 m (20–30% Q) from offices, 0–361 m (0–10% Q) from parks]*	*(0 m from agriculture, 0% cov. of office, 0% cov. of water)*
Demographical	*(Population density in [2,133–7,623] (60–70% Q))*	*(Population density in [26–55] (30–40% Q))*

**Figure 11:**
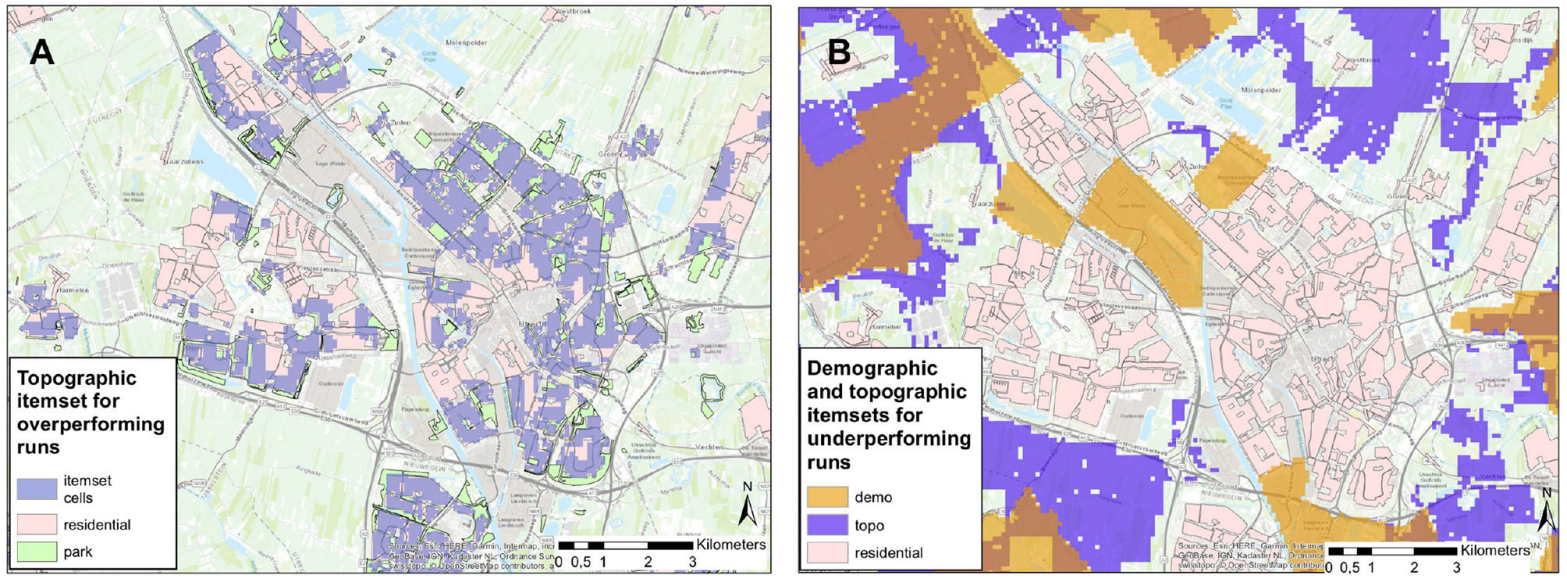
Maps for the city of Utrecht illustrating frequent topographical and demographical situations in Table 3. In **(A)**, the areas of purple correspond to a topographical situation *(0 m from residential area, 0–361 m from parks)*, which is often associated with a longer running distance. In **(B)**, the areas in purple and orange correspond to a topographical situation *(0 m from agriculture, 0% cov. of recreation, 0% cov. of residential)* and a demographical situation *(population density in [26–55])* with a shorter running distance.

Furthermore, to better interpret the extracted topographical and demographical situations, we visualized their corresponding areas on the maps and illustrated three of them in Figure 11. We realized that the locations found within topographical and demographical situations are largely corresponding to each other (purple and orange areas in Figure 11B show overlaps).

From topographical situations, we discovered that they are largely dominated by the distance to or coverage of residential and business areas (which might be related to their homes and offices), especially when such places are located close to parks. For instance, less active runners often perform above average, when they start in or nearby residential areas that are close to parks (e.g., purple areas in Figure 11A). These findings are consistent with the important role of parks in forming an attractive environment for runners (45). On the contrary, when less active people start running where certain types of green spaces (like forest, recreation, agriculture) are sparse, they tend to under-perform (e.g., purple areas in Figure 11B). From demographical situations, we observed that the combinations of demographic features are less clearly associated with a running behavior pattern. We therefore think that the demographic results should be interpreted with care and might not tell us more than what is already captured by topography.

## 4. Conclusion, Discussion, and Future Work

In this article, we presented an integrated data-driven methodology to understand the complex relationship between situations (X), running performance (Y), and individual characteristics of runners (Z). We examined this triadic relation by combining large-scale mobile application data and geographical data. This study demonstrates that the cross-linking of various data streams can deliver new insights about human behaviors in public health.

First, to answer research question 1, we found that several temporal and environmental features (i.e., “hour in a day,” “day in a week,” “temperature,” “distance to residential areas,” and “population density”) influence the running distance of people to a larger extent. Moreover, following the results in section 3.4, we answered the research question 3 by discovering certain situations that show significant associations with running frequency and distance of less active runners. For instance, a Sunday morning with moderate temperature attracts less active people to run often and far. Our findings show that the presented method is able to discover specific situations in which particular kinds of runners frequently run above or below their average distance. In contrast to previous work, we assessed a large variety of contextual situations (covering temporal, weather-related, topographical and demographical categories). Moreover, we did not only explore the influence of individual features (e.g., parks), but also took the interplay between features into account. For instance, previous studies demonstrated the importance of parks for forming an attractive running environment (37, 45). We observed that it is the combination between parks and certain locations (e.g., the parks located close to residential areas in Figure 11A) that plays a positive role on running distance. In contrast, the residential areas far away from the parks and parks far away from residential areas do not show this effect. A previous qualitative study has indicated that the nuanced interplay among contextual factors can play an important role for planning physical activities (25). Our empirical findings can help shed light on such complex effects.

Furthermore, to answer the research question 2, our results in section 3.3 indicate that the running distance of less active runners is more sensitive to selected situations (including weather, time, topographical, and demographical variables) compared to active runners. To illustrate, poor weather likely results in shorter runs for less active runners, while the running distance of active runners is less likely to be affected. This finding from the not self-reported data provided a significant empirical evidence for the complex relation between situational barriers and different human behaviors (20). Our findings are also in line with earlier research that demonstrated (perceived) situational barriers have more impact on the physical activity behavior of the individual when a habit is not established (22, 46, 47). Hence, it is important to consider situations of the individual carefully when developing future interventions to support less active runners. For instance, our results provide concrete measures of both running sustainability and environmental situations for developing an intelligent mobile system to promote physical activity. Until now, few developed mobile systems have acknowledged the importance of situations and assessed “opportunistic” situations to potentially support a certain physical activity behavior (48). Those researches defined the opportunity of situations from a theoretical perspective, without incorporating any empirical evidence. We therefore believe that the knowledge we gained in this paper adds important value to such mobile intervention systems.

Meanwhile, there are still several limitations to our analysis. First of all, a general methodological challenge concerns the fact that in this paper we extracted situations based on measured performances. Hence, we only took situations into account that were in the dataset, i.e., chosen by individuals in our sample. This means, on the one hand, that we could not make use of empirical data about situations in which people do not run (negative data). Hence, our current dataset does not allow predicting whether people start a run. This is why we concentrate in this paper on the individual's running performance. On the other hand, there might be infrequent or absent situations that are fit for running but were not chosen by the individual. Every empirical dataset that is based on behavior has to deal with this selection bias. For this reason, it may be the case that our method overlooks important running opportunities just because most people choose not to run in such situations, even though this would be beneficial. For example, the fact that not many people run within sport areas does not yet mean that such facilities do not provide excellent opportunities for running. One way to overcome this problem is to assess running opportunities based on simulations (49). For example, agent-based models (50) or optimization based simulations of runs could consider detailed geographic and temporal information without choice bias to assess the feasibility of running. Also, more detailed track information and corresponding contextual features rather than only the starting points used here could be taken into account. For example, intermediate tracking points and different aggregated running phases. In addition, although our objective was to study participants from the Netherlands, we are aware that the running behavior may vary according to cultural factors. Thus, a comparison study across different countries would be interesting in future work for verifying the findings.

Moreover, although we interpreted our findings using background knowledge and geographic maps, we are aware that indirect effects and self-selection make the interpretation challenging. The actual causal relationship between situations and user behaviors should be further examined. For example, qualitative research (such as interviews) is a promising way to complement our current method to further understand a user's choices interactively. Such studies can not only contextualize our results, but also help distinguishing causal and non-causal relations. While we only studied correlation in this work, another option is studying causal relations using other techniques, like machine learning [e.g., using causal mining to model human behaviors (51)] and statistical [e.g., structural equation modeling to handle indirect effects (52)] methods. In future work, we aim to identify situation-based rules that could be used in mobile intervention systems from our findings.

## Data Availability Statement

The mobile running dataset analyzed in this study is not publicly available, because it was collected by a commercial partner with privacy confidentiality agreements. Requests to access the dataset should be directed to MYLAPS (http://www.mylaps.com/). The geographical data about Dutch landuse and demographics are included in the article. Please refer the links of Dutch Centraal Bureau voor de Statistiek given in section 1.

## Author Contributions

SW and JT cleaned and processed the mobile running dataset, while SS collected and processed the geographical datasets. SW combined the different datasets and performed the data mining studies. SS generated the geographical maps. KS and MD provided the domain knowledge on sports and public health. BK supervised the project and provided the funding resource. SW, SS, and KS discussed the results and drafted the manuscript. All authors contributed to the manuscript revision, read, and approved the submitted version.

## Conflict of Interest

The authors declare that the research was conducted in the absence of any commercial or financial relationships that could be construed as a potential conflict of interest.

## Footnotes

^1^https://www.mylaps.com/

^2^https://www.cbs.nl/nl-nl/dossier/nederland-regionaal/geografische-data/natuur-en-milieu/bestand-bodemgebruik

^3^https://www.cbs.nl/nl-nl/dossier/nederland-regionaal/wijk-en-buurtstatistieken

^4^In Geodesy (Amersfoort/RD New projection): http://spatialreference.org/ref/epsg/amersfoort-rd-new/.

^5^Note that measuring performance in terms of relative running distance captures only one aspect of running performance, though. Other performance measures (such as speed and running intensity) could also be taken into account.

^6^https://github.com/wannesm/dtaidistance
